# Spatio-temporal activity patterns of odor-induced synchronized potentials revealed by voltage-sensitive dye imaging and intracellular recording in the antennal lobe of the cockroach

**DOI:** 10.3389/fnsys.2012.00055

**Published:** 2012-07-25

**Authors:** Hidehiro Watanabe, Hiroyuki Ai, Fumio Yokohari

**Affiliations:** Division of Biology, Department of Earth System Science, Fukuoka UniversityFukuoka, Japan

**Keywords:** olfaction, synchronized potentials, optical imaging, voltage-sensitive dye, intracellular recording, local interneurons, projection neurons, insects

## Abstract

In animals, odor qualities are represented as both spatial activity patterns of glomeruli and temporal patterns of synchronized oscillatory signals in the primary olfactory centers. By optical imaging of a voltage-sensitive dye (VSD) and intracellular recording from secondary olfactory interneurons, we examined possible neural correlates of the spatial and temporal odor representations in the primary olfactory center, the antennal lobe (AL), of the cockroach *Periplaneta americana*. Voltage-sensitive dye imaging revealed that all used odorants induced odor-specific temporal patterns of depolarizing potentials in specific combinations of anterior glomeruli of the AL. The depolarizing potentials evoked by different odorants were temporally synchronized across glomeruli and were termed “synchronized potentials.” These observations suggest that odor qualities are represented by spatio-temporal activity patterns of the synchronized potentials across glomeruli. We also performed intracellular recordings and stainings from secondary olfactory interneurons, namely projection neurons and local interneurons. We analyzed the temporal structures of enanthic acid-induced action potentials of secondary olfactory interneurons using simultaneous paired intracellular recording from two given neurons. Our results indicated that the multiple local interneurons synchronously fired in response to the olfactory stimulus. In addition, all stained enanthic acid-responsive projection neurons exhibited dendritic arborizations within the glomeruli where the synchronized potentials were evoked. Since multiple local interneurons are known to synapse to a projection neuron in each glomerulus in the cockroach AL, converging inputs from local interneurons to the projection neurons appear to contribute the odorant specific spatio-temporal activity patterns of the synchronized potentials.

## Introduction

The primary olfactory centers, which consist of the olfactory bulb in mammals and the antennal lobe (AL) in insects, are compartmentalized into a large number of spheroidal glomeruli (Hildebrand and Shepherd, 1997). Each glomerulus is a neuropil where a large number of olfactory receptor neurons synapse onto a smaller number of secondary interneurons. Genetic studies indicate that many olfactory receptor neurons expressing the same type of odorant receptor protein all converge onto a single glomerulus in fruit flies, or onto a few glomeruli in rodents (Mombaerts et al., 1996; Couto et al., 2005; Luo and Flanagan, 2007). Since each olfactory receptor neuron responds to a range of odorants sharing similar molecular features, each glomerulus must be involved in the processing of several odorants (Fujimura et al., 1991; Couto et al., 2005; Luo and Flanagan, 2007). Optical imaging studies using a calcium-sensitive dye have revealed that each odorant induces specific spatially defined combinatory patterns of glomerular activity (Galizia et al., 1999; Rubin and Katz, 1999; Sachse et al., 1999; Sachse and Galizia, 2002; Wang et al., 2003; Soucy et al., 2009). In addition, olfactory stimuli are known to induce synchronized spiking of the neural populations in both the olfactory bulb and the AL, which highly correlate with oscillation of their local field potentials (Laurent and Davidowitz, 1994; Laurent et al., 1996; MacLeod and Laurent, 1996; Lam et al., 2000; Christensen et al., 2003). In locusts, intrinsic neurons of the higher olfactory center, mushroom body, detect the odor-induced synchronized action potentials of output neurons of the AL, projection neurons, which are phase-locked to the oscillations of the local field potential (MacLeod et al., 1998; Perez-Orive et al., 2002, 2004). Different odorants evoke specific temporal activity patterns of local field potentials in locusts (Laurent et al., 1996) and fruit flies (Tanaka et al., 2009). Therefore, odor identities are represented by both spatial and temporal activity patterns in the specific neural assemblies of the primary olfactory center. The insect AL, in which it is possible to track neural events within a network of fully characterized neurons, may provide a particularly valuable model system for examining the neural basis of odor representation.

Optical imaging and recording of local field potentials combined with pharmacological treatments have revealed important roles of inhibitory networks in relation to the spatial and temporal odor representations. Optically recording the calcium responses from selectively stained projection neurons in the honeybee AL have been revealed that the number of glomeruli that showed excitatory responses to an odor is increased following inhibition of GABA receptors (Sachse and Galizia, 2002). In addition, inhibition of GABA receptors abolishes both the local field potential oscillation and the synchronization of the odor-coding assemblies of projection neurons in locusts (MacLeod and Laurent, 1996) and fruit flies (Wilson and Laurent, 2005; Tanaka et al., 2009). In addition, honeybees are unable to finely discriminate odorants following inhibition of the GABA receptor (Stopfer et al., 1997). Therefore, GABAergic neurons in the AL, acting as local interneurons, may be involved in both spatial and temporal representations of odor identities.

The goal of the current study is to reveal the neural basis of the spatial and temporal representations of odor qualities in the insect AL. To address this question, we developed an optical imaging technique using a voltage-sensitive dye (VSD) in the AL of the cockroach, *Periplaneta americana*. In the cockroach AL, 205 unambiguously identified glomeruli are divided into two major groups: the antero-dorsal and the postero-ventral groups (Salecker and Boeckh, 1996; Nishino and Mizunami, 2006; Watanabe et al., 2010, 2012; Nishino et al., 2012). The antero-dorsal group consists of small oval-shaped glomeruli that receive sensory inputs from antennal perforated basiconic sensilla, whereas the postero-ventral group is composed of variously-shaped large glomeruli receiving sensory inputs from trichoid and grooved basiconic sensilla of the antenna (Nishino and Mizunami, 2006; Watanabe et al., 2010, 2012). Using VSD imaging, we simultaneously monitored the odor-driven depolarizing potentials evoked in the glomeruli of both the antero-dorsal and the postero-ventral groups with high temporal resolution. We observed odor-specific temporal patterns of depolarizing potentials evoked in odor-specific combinations of glomeruli in the AL. Since glomeruli activated by enanthic acid were able to identify correctly using a topological map of glomeruli based on the projection patterns of 10 sensory tracts in the cockroach AL (Watanabe et al., 2010), we attempt to compare the odor-induced temporal activity patterns of glomeruli observed in VSD imaging with the activity patterns of single olfactory interneurons arborizing in the glomeruli. Our results indicate that VSD imaging appears to detect the odor-induced synchronized firing of multiple local interneurons. The coordinated activities of local interneurons could play a critical role in the generation of the spatial and temporal activity patterns of glomeruli.

## Materials and methods

### Preparation

**Figure 1:**
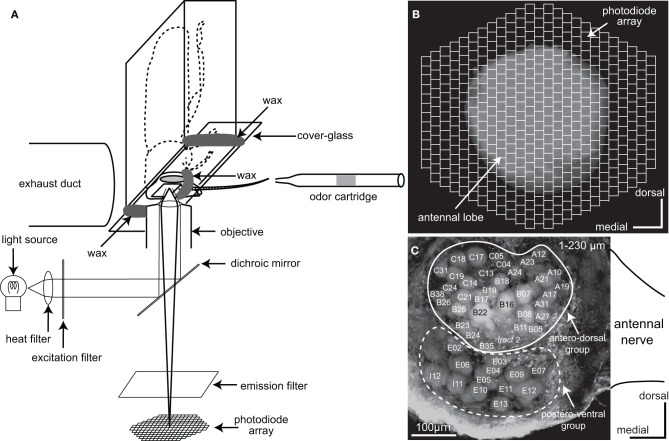
**Optical imaging of a voltage-sensitive dye (VSD) in the cockroach AL. (A)** A schematic diagram of VSD imaging from the cockroach AL. The cockroach AL was illuminated using a metal halide lamp. The incident light passed through a heat filter and a 535 ± 25 nm band-pass excitation filter and was reflected onto the preparation by a dichroic mirror. The odor cartridge is positioned at 10 mm away from the tip of the antenna ipsilateral to the recorded AL. Olfactory stimuli were delivered along the whole antenna. **(B)** The image of the preparation was formed onto a 454-element photodiode array after passing through a 615 nm long-pass emission filter. Each photodiode corresponds to a 25 × 25 μm area of the cockroach AL and the 454-element photodiode array covered the whole anterior surface of the cockroach AL. **(C)** Reconstructed confocal image of the cockroach AL (230 μm image stack). In this study VSD imaging simultaneously recorded changes of the membrane potentials in both the antero-dorsal and the postero-ventral groups of glomeruli located just beneath the anterior surface of the AL. The nomenclatures of the glomeruli and the sensory tracts are from Watanabe et al. (2010). Glomeruli surrounded by solid and broken lines belong to the antero-dorsal and the postero-ventral groups, respectively.

Male adult cockroaches, *Periplaneta americana*, were obtained from laboratory colonies maintained under a 12:12 h light:dark cycle at 28°C in Fukuoka University. Prior to both optical imaging and electrophysiological recording, cockroaches were anesthetized with ice and attached to the experimental chambers with low-melting point wax. To prevent the brain from being moved by antennal moving, both antennae were also fixed to the chamber with wax (Figure 1A). The head capsule was opened between the antennae and the ocelli and most of the muscles and tracheae above the brain were removed.

### Voltage-sensitive dye imaging

For VSD imaging, the brain was desheathed with fine tweezers, stained with a saline solution containing 3 mg/ml RH414 (Invitrogen, Eugene, Oregon, USA) for about 30 min, and then washed with the saline to remove excess dye. The composition of the saline solution for VSD imaging was: NaCl 124 mM, KCl 10 mM, CaCl_2_ 20 mM, MgCl_2_ 8 mM, 3-morpholinopropanesulfonic acid (MOPS) 3 mM, MOPS sodium salt 3.9 mM, sucrose 40 mM (pH 6.5 adjusted with HCl) (Ai and Inouchi, 1996). The head was then placed anterior-side-down onto the stage of an inverted microscope (Axiovert 100, Carl Zeiss, Germany) and fixed with wax to a custom glass chamber, with a cover-glass on the ventral side of the head (Figure 1A). The AL was positioned in the center of the field of the objective lens (Plan-Apochromat 10 × 0.45 NA). The camera unit of the optical recording system (Neuroplex, RedshirtImaging, Co. Ltd., USA) was equipped with an array of 454 photodiodes (Figure 1B). Each photodiode corresponded to a tissue area of 25 × 25 μm. Excitation and emission filters in the optical system passed light of wavelengths 535 ± 25 nm and >615 nm, respectively, which were separated using a dichroic mirror placed in the light path of metal halide lamps (KMH-250, BMH-250; Kiyohara Optical Laboratory, Japan). The VSD signals were simultaneously acquired from all photodiodes at 6 ms per frame. Before commencing optical recording, the z-axis of the stage was adjusted using the focusing drive to coincide with the plane through the surface of the AL. Signals evoked by the olfactory stimuli were then recorded at a series of z-axis levels and the position of the z-axis of the stage was finally set to the plane at which acquired depolarizing potentials were most conspicuous. The responses to olfactory stimuli were analyzed in the areas where depolarizing potentials were repeatedly produced across trials. In this study, the changes of membrane potentials were represented as changes in the ratio of fluorescent intensities, i.e., (*F*_0_–*F*_*t*_)/*F*_0_. *F*_*t*_ was the fluorescent intensity at a certain time (t) and *F*_0_ was the averaged fluorescent intensity during a 500 ms period before odor stimulation. In our VSD imaging system could not store the potential data in an analyzable format, such as a text files. Therefore, we manually calculated the time distributions of depolarizing potentials from JPEG images.

### Single and paired intracellular recording and staining

The methods used for intracellular recording from, and staining of, individual secondary olfactory interneurons of the cockroach were modified from those reported in previous studies (Nishino et al., 2003, 2011, 2012). After the brain sheath had been slightly softened by Actinase E (Kaken Seiyaku, Tokyo, Japan), the brain and platinum ground electrode were immersed in a cockroach saline solution used for intracellular recording (Yamasaki and Narahashi, 1959; NaCl 210.2 mM, KCl 3.1 mM, CaCl_2_ 1.8 mM, NaH_2_PO_4_ 0.2 mM, Na_2_HPO_4_ 1.8 mM, pH 7.2). To stabilize the brain, a glass rod was inserted into the cavity formed by removal of the esophagus.

A borosilicate glass microelectrode pulled by a laser puller (P-2000; Sutter Instruments, Novato, CA, USA) was filled with 8% Lucifer Yellow (Sigma, St. Louis, USA) or 10 mM Alexa 647 (Invitrogen, Eugene, Oregon, USA) in 1 M LiCl (aqueous). An electrode was inserted into the cluster of somata of secondary olfactory interneurons located in the dorsal region of the AL (Distler and Boeckh, 1997a,b). In simultaneous paired intracellular recordings from two distinct secondary olfactory interneurons, two electrodes filled with different fluorescent dyes (Lucifer Yellow and Alexa 647) were inserted into the soma cluster of the ipsilateral AL. Neural activities of individual neurons were amplified (MEZ8301, Nihon Kohden, Tokyo, Japan) and displayed on an oscilloscope and a PowerLab data acquisition system (AD Instuments Japan Inc., Nagoya, Japan). After acquisition of the olfactory responses, the neuron was filled with fluorescent dye by injecting a hyperpolarizing current. Immediately after intracellular dye filling, anterograde staining of antennal afferents were performed as follows (Watanabe et al., 2010; Nishino et al., 2011, 2012). The antennal nerve on the side ipsilateral to the recording site was exposed and cut at the flagellar base. The proximal cut end of the antennal nerve was inserted into a tapered glass capillary filled with a 10% aqueous solution of micro-ruby (dextran tetramethyl rhodamine with biotin, 3000 MW, D-7162, Invitrogen, Eugene, Oregon, USA). The cut stump of the antennal nerve was kept in contact with the dye in a humid chamber at 4°C overnight. Subsequently, the double- or triple-stained brain was dissected out from the head capsule. The isolated brain was fixed with 4% formaldehyde solution at 4°C for 3–5 h, dehydrated in an ascending ethanol series (from 70 to 100%), and then cleared in methyl salicylate.

### Olfactory stimulation

In the cockroach, most of the olfactory receptor neurons that respond to general odors have been classified into eight groups on the basis of similarities of response spectra (Fujimura et al., 1991). For VSD imaging, five odorants which had distinctive excitatory effects on any of the receptor cell groups were selected: citral, terpineol, pentanol, octanol, and enanthic acid. Pure solutions of each odorant were diluted with ethanol to produce odorant vapor pressures of 0.01 mmHg at 25°C, and a small piece of filter paper soaked with 50 μL of one of the solutions was inserted into each cartridge (Figure 1A). The odorant vapor pressures were estimated from the Antoine or Claperyon-Clausius equation using their saturated vapor pressure value. Five odorants induced consistent responses on VSD imaging (See “Results”). In this study, spatio-temporal patterns of the VSD signal evoked by pentanol, citral, and enanthic acid were analyzed in detail. We could not accurately identify the glomeruli in which the VSD signals were evoked by tested odorants except for enanthic acid (See “Results”). Therefore, for identification of the signal sources of VSD imaging, enanthic acid was used as an odorant in single and paired intracellular recordings of activities of secondary olfactory interneurons.

Fresh air for the olfactory stimuli and for control was taken from outdoors via a diaphragm pump. The air was passed through a cotton filter, a charcoal filter, and then a silicone tube (internal diameter 5 mm). The flow of air within the tube was maintained at about 1 L/min using a flowmeter. Using three-way valves, the tube was further divided into six tubes which were connected to five cartridges containing different odorants (50 μL of odor solution absorbed onto a small piece of filter paper) and a blank cartridge. Odorant cartridges and a blank (control) cartridge were arbitrarily selected for stimulation by manual operation of the valves. To stimulate whole antennal segments, the tip of cartridge was positioned about 10 mm apart from the tip of antenna, and air from around the preparation was continuously exhausted by a duct located behind the base of antenna (Figure 1A). The three-way solenoid valve was controlled by the stimulator (SEN7203, Nihon Kohden, Tokyo, Japan). The stimulus period was set at 2 s for VSD imaging. In intracellular recording, we focused mainly on the enanthic acid-induced phasic on-response of VSD signals (See “Results”). In addition, long-duration recordings were difficult in intracellular recording. Therefore, the stimulus period was set at 1 s for intracellular recording. Each odorant was presented two or three times. We presented five different odorants in sequence with 1 min interval. After first set of odor stimuli, we presented five odorants again in a different order in VSD imaging. Therefore, the time distance between first and second trials of a given odorant is more than 5 min. When we change the odorants, a new cartridge including another odorant was moved at the same position as the previous cartridge.

### Confocal microscopic observation

The cleared brains were observed with a confocal laser scanning microscope (LSM-510; Carl Zeiss, Jena, Germany) equipped with argon, HeNe 1, and HeNe 2 lasers. Single neurons labeled with Lucifer Yellow or with Alexa 647 were visualized using the argon laser with a band-pass emission filter (505–530 nm) or with the HeNe 2 laser with a long-pass filter (>650 nm), respectively. Glomerular structures labeled with micro-ruby or with RH414 were visualized using the HeNe 1 laser with a band-pass filter (560–615 nm) or with a long-pass filter (>560 nm), respectively. Two different objective lenses, plan-apochromat 10 × 0.8 NA and 20 × 0.8 NA, were used for LSM observation. Optical sections were usually made at a resolution of 1024 × 1024 pixels/inch at 6 μm intervals throughout the whole brain and at 3 μm intervals throughout the glomerular part of the AL. Acquired optical image files were converted to TIFF-formatted files with the software LSM Image Browser (Carl Zeiss, Jena, Germany). The contrast and brightness of all figures presented here were adjusted appropriately using Adobe Photoshop CS3 and Illustrator CS3 (Adobe Inc., CA, USA).

### Terminology

In the cockroach AL, all glomeruli were unambiguously identified on the basis of the innervation patterns of the 10 sensory tracts in addition to their shapes, sizes, and locations (Watanabe et al., 2010). In VSD imaging, to identify the glomeruli and glomerular groups where the depolarizing potentials were evoked, the VSD fluorescent images of the AL were superimposed on confocal images acquired in the plane in which the VSD signals were recorded in the same preparations (Figure 1C). We could correctly identify the glomeruli, activated by enanthic acid (see “Results” section), but not glomeruli activated by the other odorants because the pixels were larger than the corresponding glomeruli. As the innervation patterns of the 10 sensory tracts to the glomeruli were visualized by the anterograde staining of antennal afferents, the glomeruli innervated by the intracellularly recorded olfactory interneurons could be identified unambiguously by helps of the glomerular map of the AL (Watanabe et al., 2010). The identifications of glomeruli in the two different experiments were performed independently by two different experimenters. The nomenclatures and detailed morphological features of glomeruli and glomerular groups were described in our previous studies (Watanabe et al., 2010, 2012). The orientation and position of the brain is shown with reference to the body axis.

### Data analysis

We evaluated the temporal dynamics in enanthic acid-induced action potentials of secondary olfactory interneurons using programs attached to PowerLab and Microsoft Excel software. The instantaneous spike frequencies (Hz) were defined as reciprocals of the time intervals between successive spikes. Since VSD imaging acquired the averaged membrane potential during the 6 ms period of each frame, we counted spike numbers every 6 ms in intracellular recordings and made spike histograms. The spike histograms were created from neural activities for a period of 500 ms from the onset of enanthic acid stimulation. 202 spikes obtained from 16 responses of 6 projection neurons and 131 spikes obtained from 32 responses of 15 local interneurons were used in our analysis. Power spectral densities of each of olfactory interneurons were calculated every 10 Hz (Figure 8C) or 1 Hz (Figure 8D) by a program attached PowerLab data acquisition system. To evaluate frequency characteristics of membrane potentials during olfactory stimulus, the power spectral density of the discrete bin was calculated by subtracting those obtained in the peri-stimulus epoch (–500 to 0 ms) from those obtained in response epoch to enanthic acid (0–500 ms). The means and standard errors of power spectral densities obtained from 16 responses of 6 projection neurons and 32 responses of 15 local interneurons were, respectively, calculated. The averaged power spectral densities of the enanthic acid-induced neural activities in projection neurons were statistically compared with those in local interneurons in the range of 12–18 Hz (Welch's *t*-test). The comparisons of averaged power spectra were modified from the analysis of odor-induced local field potentials in the fruit fly (Tanaka et al., 2009) and of odor-induced VSD signals in the turtle (Zochowski and Cohen, 2005).

## Results

### Voltage-sensitive dye imaging from the cockroach AL

Using VSD optical imaging, we simultaneously recorded odor-induced changes in membrane potentials in the 454 pixels of the photodiodes (each pixel 25 × 25 μ m) covering the anterior surface of whole AL and the proximal region of the antennal nerve. In each preparation, the changes in depolarizing potential could be simultaneously recorded in up to 47 glomeruli (Figure 1C). Of these, 34 glomeruli (Figure 1C, surrounded by a white line) belonged to the antero-dorsal group and the remaining 13 glomeruli (Figure 1C, surrounded by a broken line) belonged to the postero-ventral group.

**Figure 2:**
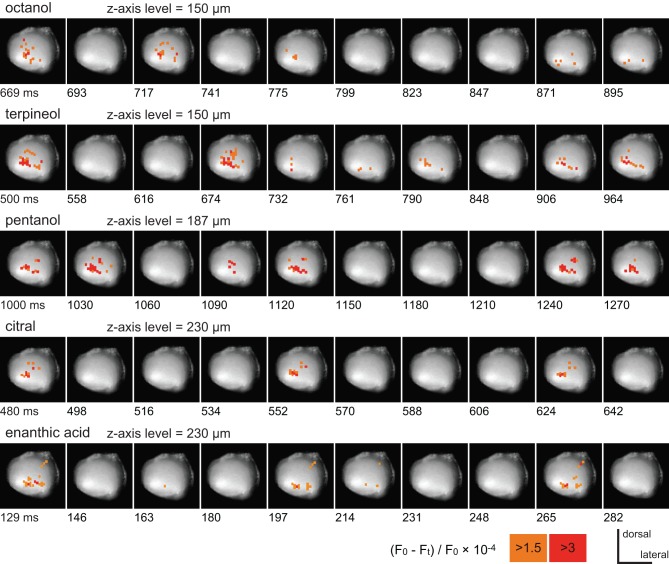
**The time courses of VSD signals induced by five different odorants.** All records were obtained from the same preparation. The pseudocolor which represent the strength of VSD signals are overlaid on each image of the antennal lobe. To show the most salient features of spatial and temporal patterns of odor-induced depolarizing potentials, the start times and intervals of serial images were arbitrary selected in each record. The position of the z-axis of the stage was set to the plane at which the acquired potentials were most conspicuous, and shown as the depth from the surface of the AL in these and subsequent figures (Figures 3, 4). The times after onset of the odor stimuli are shown at lower left of each image. The VSD signals are represented as changes in the ratio of fluorescent intensities [(*F*_0_–*F*_*t*_)/*F*_0_× 10^−4^; see “Materials and Methods”], and pixels where the signals are larger than 3 and 1.5 are labeled in red and orange, respectively.

**Figure 3:**
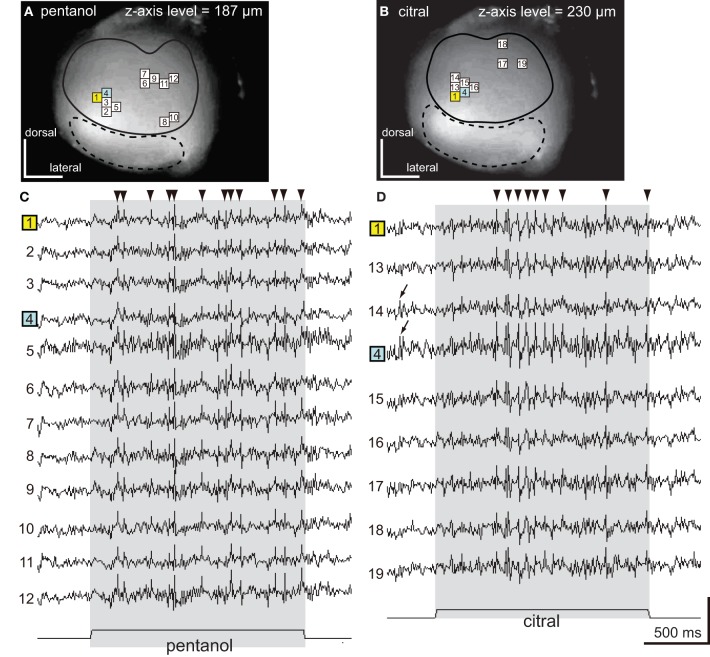
**The spatio-temporal patterns of synchronized potentials induced by pentanol and citral. (A,B)** VSD images of the cockroach AL. The depolarizing potentials induced by pentanol **(A)** and citral **(B)** were observed in 12 (numbers 1–12) and nine pixels (numbers 1, 4, 13–19), respectively, located in the antero-dorsal region of the AL. Areas surrounded by solid and broken lines correspond to the antero-dorsal and postero-ventral glomerular groups, respectively. **(C,D)** Odor-induced synchronized potentials. The pentanol- **(C)** and citral-induced **(D)** depolarizing potentials evoked in the numbered pixels shown on **(A)** and **(B)**, respectively. The number 1 (yellow pixel) and 4 (blue pixel) pixels were activated by both pentanol **(A,C)** and citral **(B,D)**. The timings of depolarizing potentials induced by each odorant were temporally synchronized across pixels (“synchronized potentials”). On the other hand, the depolarizing potentials during pre-stimulus period were not synchronized across pixels (Figure 3D; black arrows). The synchronized potentials induced by pentanol and citral persisted during 2 s of the olfactory stimulations (shaded phases). The changes of membrane potentials are shown as changes of the ratio of fluorescent intensities [See “Materials and Methods,” vertical scale bar; (*F*_0_–*F*_*t*_)/*F*_0_ × 10^−3^]. Black triangles were marked on synchronized potentials which are larger than 0.2 in these and subsequent figure (Figures 4, 5).

**Figure 4:**
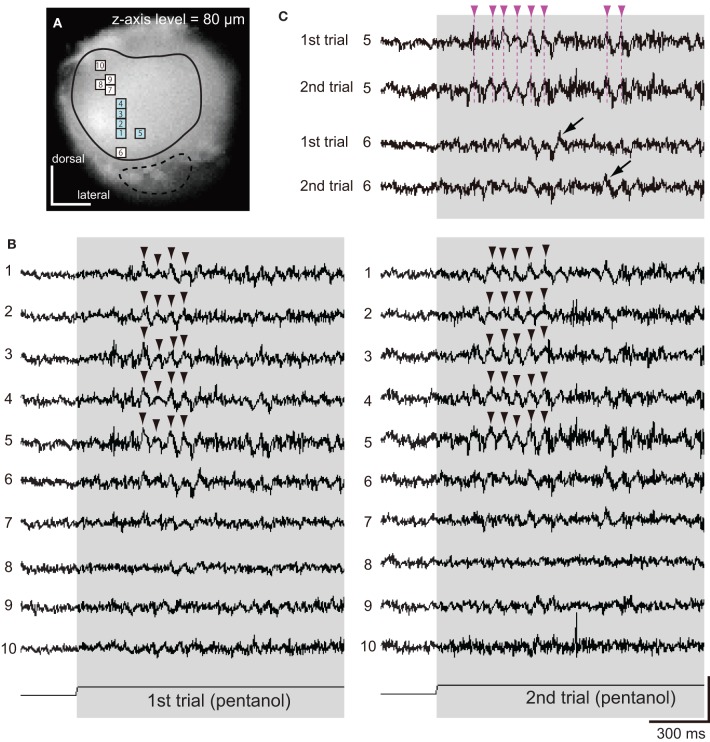
**Spatio-temporal patterns of synchronized potentials during first and second trials of pentanol stimulations. (A)** A VSD image of the cockroach AL. We observed pentanol-induced changes of membrane potentials in 10 pixels. Areas surrounded by solid and broken lines correspond to glomeruli of the antero-dorsal and postero-ventral groups, respectively. **(B)** Pentanol-induced changes of membrane potentials in 10 pixels shown on **(A)**. In both first (left) and second (right) trials, pentanol (shaded bar) generated synchronized potentials (arrow heads) in five pixels (numbers 1–5, blue pixels in A), but not in the remaining five pixels (numbers 6–10). **(C)** Changes of membrane potentials during first and second trials of pentanol stimulations in two pixels (numbers 5 and 6). In pixel 5, the peaks of pentanol-induced synchronized potentials during first and second trials were temporally coincident (broken magenta line). In pixel 6, where pentanol did not generate synchronized potentials, timings of depolarizing potentials (black arrows) did not coincident between trials. The changes of membrane potentials are shown as changes of the ratio of fluorescent intensities [vertical scale bar; (*F*_0_–*F*_*t*_)/*F*_0_ × 10^−3^].

**Figure 5:**
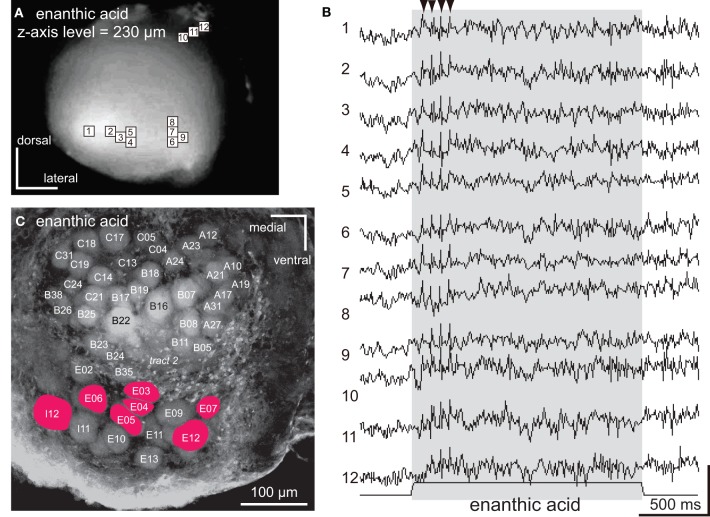
**The spatio-temporal patterns of synchronized potentials induced by enanthic acid. (A)** A VSD image of the cockroach AL. The enanthic acid-induced depolarizing potentials were observed in nine pixels (numbers 1–9) located in the postero-ventral region of the AL and three pixels (numbers 10–12) located in the dorsal soma cluster of secondary olfactory interneurons. **(B)** The enanthic acid-induced synchronized potentials evoked in 12 pixels shown in **(A)**. Enanthic acid (shaded area) induced phasic on-response of synchronized potentials. The changes of membrane potentials are shown as the changes of the ratio of florescent intensities [vertical scale bar; (*F*_0_–*F*_*t*_)/*F*_0_ × 7 × 10^−4^]. **(C)** A fluorescent image of the recorded AL stained by voltage-sensitive dye. The glomeruli where enanthic acid-induced synchronized potentials evoked were labeled in red. By superimposing the VSD image **(A)** on the confocal image **(C)** obtained from a same specimen, glomeruli (colored red) corresponding to the nine pixels (numbered 1–9) were identified and the remaining three pixels (numbered 10–12) were located in the dorsal soma clusters of secondary olfactory interneurons.

Five different odorants (octanol, terpineol, pentanol, citral, and enanthic acid) induced different spatial patterns of depolarizing potentials in the same preparation (Figure 2). The spatial distribution patterns of depolarizing potentials induced by each odorant did not changed during the stimulation. In addition, odor-induced depolarizing potentials were temporally synchronized across pixels. Therefore, we termed these depolarizing potentials observed by VSD imaging as “synchronized potentials.” The temporal structures of synchronized potentials during olfactory stimulations were also odorant specific. For example, synchronized potentials generated every 72 and 68 ms in response to citral and enanthic acid, respectively, (Figure 2). None of the odorants induced synchronized potentials in pixels corresponding to the antennal nerve. The odor-specific features of synchronized potentials were reproducibly observed across individuals (*N* = 4). Since three odorants (pentanol, citral, and enanthic acid) especially evoked conspicuous activities in odor-specific regions of the AL, we used them for further analyses of spatial and temporal activity pattern of synchronized potential (Figures 3–5).

Pentanol and citral, respectively, induced synchronized potentials in 12 and 9 pixels corresponding to some of the glomeruli in the antero-dorsal group (Figures 3A,B). Since depolarizing potentials were not synchronized across pixels during pre-stimulus period (Figure 3D; arrows), the synchronized potentials were induced by olfactory stimulations. We could not accurately identify glomeruli in which the synchronized potentials were generated because the dimensions of these glomeruli were smaller than one pixel. However, spatial patterns of pixels activated by the two odorants were significantly different (Figures 3A,B). Two pixels (numbers 1 and 4 in Figures 3A,B) were activated by both pentanol and citral but the temporal patterns of the odor-induced synchronized potentials were clearly distinct (Figures 3C,D; black triangles). Synchronized potentials were induced by citral about 500 ms after the onset of the olfactory stimulation (Figure 3D), whereas pentanol-induced synchronized potentials occurred with shorter latency (about 150 ms) (Figure 3C). During olfactory stimulations, in both cases, synchronized potentials persisted and two or three different amplitudes of synchronized potentials were generated.

Next, using different specimen, we examined spatial and temporal patterns of synchronized potentials during first and second trials of pentanol stimulations (Figure 4). The interval between first and second trials was more than 5 min, and second trials were performed after exposures of different odorants (see “Materials and Methods”). We observed odor-induced changes of membrane potentials in 10 pixels (Figure 4A). In both trials, pentanol-induced synchronized potentials were generated in five pixels (numbers 1–5 in Figures 4A,B), whereas synchronized potentials were not evoked in the remaining five pixels (numbers 6–10 in Figures 4A,B). In each pixel of the former group, the timings of synchronized potentials during second trial corresponded approximately to those during first trial (magenta arrowheads in Figure 4C). In each pixel of the later group, however, timings of depolarizing potentials were not correlated between trials (black arrows in Figure 4C). These results suggested that odor-induced spatio-temporal patterns of synchronized potentials did not change between first and second trials of the same odor stimulations.

The synchronized potentials induced by enanthic acid were observed in 12 of 454 pixels (Figures 5A,B). To identify the glomeruli in which the synchronized potentials were generated, the VSD images were superimposed on the confocal stack of images from the same AL preparation. Nine of the 12 pixels corresponded to seven glomeruli of the postero-ventral group (E03, E04, E05, E06, E07, E12, and I12 glomeruli in Figure 5C) and the remaining three pixels (numbers 10, 11, and 12 in Figure 5A) were located in the anterior soma cluster, comprising the somata of the secondary olfactory interneurons (Distler and Boeckh, 1997a,b; Distler et al., 1998). In contrast to the longer decay phases of synchronized potentials induced by citral and pentanol, the potentials induced by enanthic acid persisted for only 150–500 ms after the onset of the olfactory stimulus (Figure 5B). In each glomerulus (pixel numbers 1–9), the amplitudes of synchronized potentials stabilized during enanthic acid stimulation. Thus, three distinct odorants (pentanol, citral, and enanthic acid) induced both spatially and temporally distinct activity patterns of synchronized potentials. These results strongly suggest that odor-identities are encoded by spatio-temporal patterns of synchronized potentials.

### Intracellular recording from single secondary olfactory interneurons

**Figure 6:**
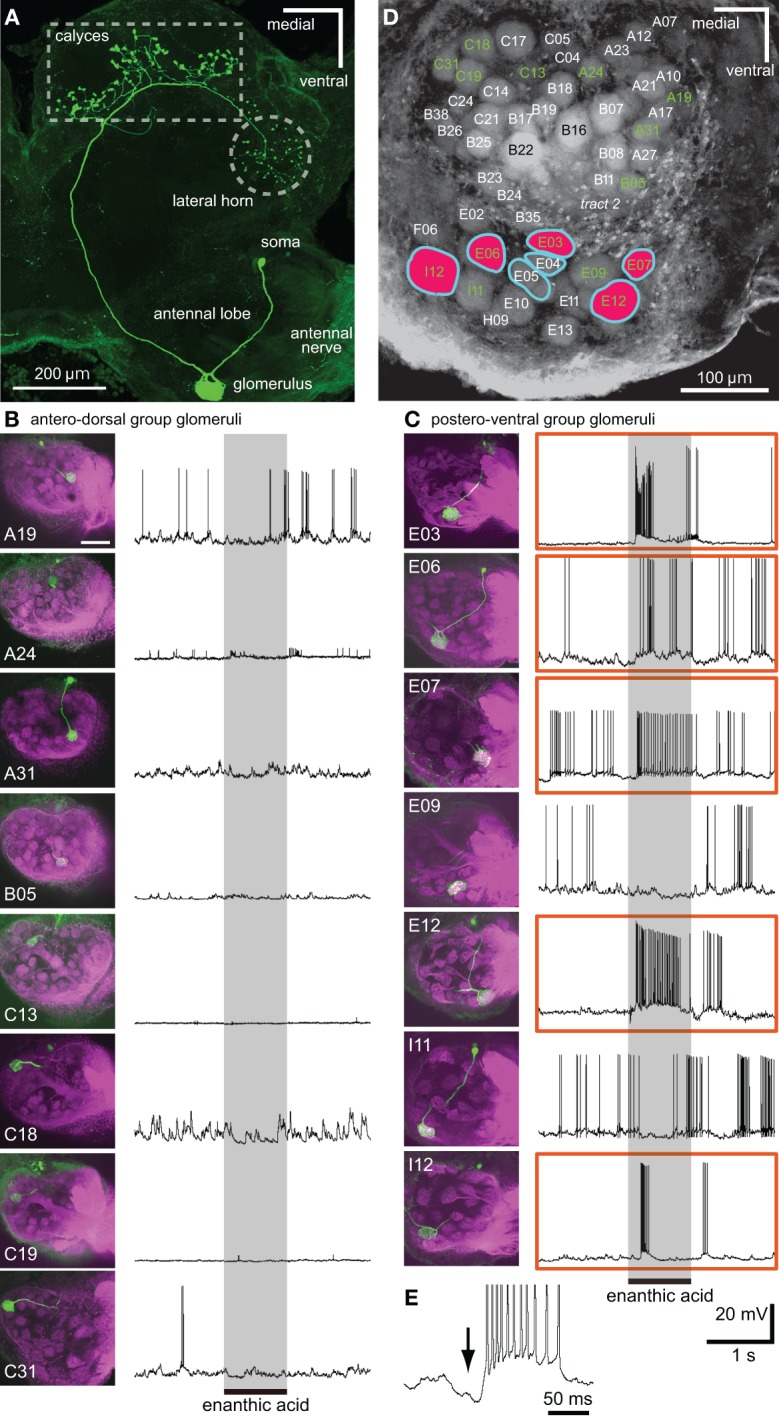
**Single intracellular recordings from projection neurons arborizing in single glomeruli located just beneath the anterior surface of the AL. (A)** Morphology of a uniglomerular projection neuron with dendrites in a glomerulus (E06 glomerulus). The axon is projected to the calyces of the mushroom body and to the lateral horn via the medial antenno-protocerebral tract. **(B,C)** Somata and dendrites of 15 projection neurons (shown in green in left images) and their responses to enanthic acid (right traces). Eight projection neurons arborize in single glomeruli of the antero-dorsal group **(B)**, and the remaining seven projection neurons innervate single glomeruli of the postero-ventral group **(C)**. In each of the left images, the sensory afferents are shown in magenta and the names of glomeruli are also shown. Five projection neurons, which have dendritic arborizations in E03, 06, 07, 12, and I12 glomeruli (enclosed by orange boxes), exhibited strong excitatory responses to enanthic acid stimulation (shaded areas). Scale bar = 100 μm in **B** and **C** (see upper left image). **(D)** Summary of the spatial pattern of enanthic-acid responsive glomeruli. The names of 15 glomeruli innervated by intracellularly recorded projection neurons are labeled in green. Five enanthic acid-responsive projection neurons arborize in the glomeruli colored red. In VSD imaging, enanthic acid-induced synchronized potentials in the seven glomeruli surrounded by light blue lines (see Figure 5). **(E)** Enanthic acid-induced hyperpolarizing membrane potential (arrow) before the depolarizing phase recorded from a uniglomerular projection neuron with dendrites in I12 glomerulus.

**Figure 7:**
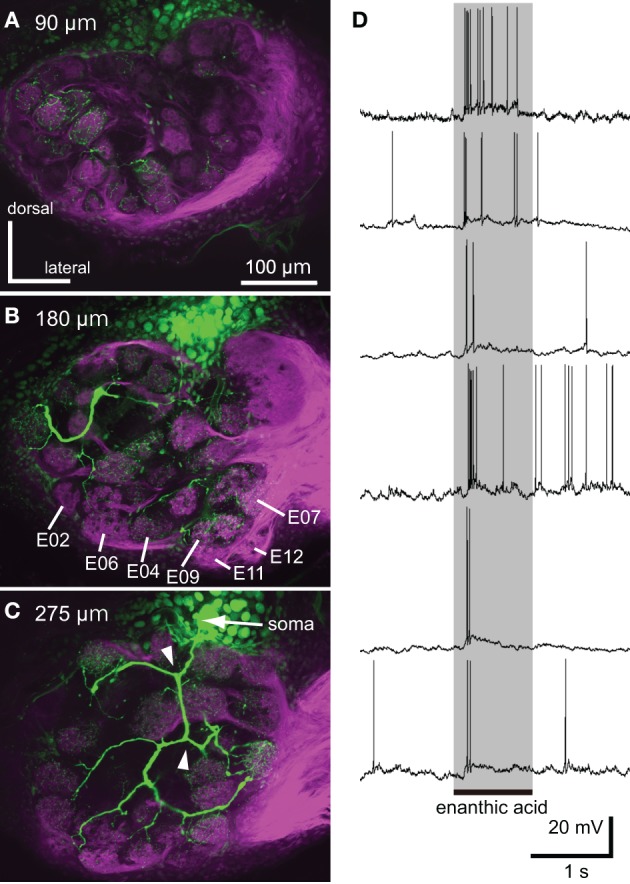
**Neural activities of single local interneuron innervating multiple glomeruli. (A–C)** Serial optical sections, from anterior to posterior, of a typical enanthic acid-responsive local interneuron. The depths from the anterior surface of the AL are shown in upper left. The local interneuron innervates most, but not all, glomeruli in the cockroach AL (green). The neuron innervated the glomeruli in which enanthic acid-induced synchronized potentials were evoked (**B**, Figure 5). The axons give rise to y-shaped branches (arrowheads in **C**), which have been identified as GABAergic local interneurons (Husch et al., 2009b). Magenta: sensory afferents. **(D)** Phasic excitatory on-responses of local interneurons to enanthic acid. We recorded from 38 oligoglomerular local interneurons and 15 of them were enanthic acid-responsive. Six typical local interneurons innervated different sets of glomeruli exhibited phasic on-responses to enanthic acid stimulation (shaded phase). Similar response patterns were observed in all local interneurons that were recorded from.

Three pixels (numbers 10, 11, and 12 in Figure 5B), which recorded enanthic-acid induced synchronized potentials, corresponded to the cluster of somata of projection neurons and local interneurons (Distler and Boeckh, 1997a,b; Distler et al., 1998). In order to clarify which interneurons contributed to the synchronized potentials, we intracellularly recorded responses to enanthic acid from single projection neurons (Figure 6) and local interneurons (Figure 7) which had dendritic arborizations in the glomeruli located just beneath the anterior surface of the AL.

By intracellular stainings from single olfactory interneurons, 15 different projection neurons arborizing in single glomeruli located in the anterior region of the AL were morphologically characterized (Figure 6). Axons of these projection neurons ran the medial antenno-protocerebral tract and terminated both in the ipsilateral mushroom body calyces and in the lateral horn (Figure 6A). Eight projection neurons had dendritic arborizations in glomeruli of the antero-dorsal group (Figure 6B) and the remaining seven arborized in glomeruli of the postero-ventral group (Figure 6C). Five projection neurons arborizing in the five glomeruli (E03, E06, E07, E12, and I12 glomeruli in Figures 6C,D) of the postero-ventral group exhibited strong excitatory responses to enanthic acid stimulation (Figure 6C). All of these five glomeruli corresponded to glomeruli in which enanthic acid-induced synchronized potentials were recorded by VSD imaging (Figure 6D). Therefore, if VSD imaging detects the depolarizing potentials of projection neurons, enanthic acid must evoke synchronized phasic on-responses in the projection neurons despite innervating different glomeruli. However, temporal activity patterns of enanthic acid-induced spike arrays varied according to the innervated glomeruli. Projection neurons having arborizations in E03 and I12 glomeruli showed phasic excitatory on-responses to enanthic acid whereas those innervating E06, E07, and E12 glomeruli exhibited longer decay responses to the odor (Figure 6C). In addition, most projection neurons exhibited phasic hyperpolarizing potentials before the depolarizing phase (black arrow in Figure 6E), which suggests that these projection neurons receive phasic inhibitory inputs from other neural elements, such as GABAergic local interneurons.

We succeeded in intracellular recordings and stainings from 38 single local interneurons (Figure 7). All characterized local interneurons exhibited oligoglomerular projections; they had dendritic arborizations in most, but not all, glomeruli (Figures 7A–C) and their primary axons formed y-shaped branches in the AL (Figure 7C, arrowheads). These morphological features are consistent with those of GABAergic type-1 local interneurons, which make inhibitory synapse with the projection neurons (Distler et al., 1998; Husch et al., 2009b). All stained local interneurons extended neurites into the glomeruli in which synchronized potentials were evoked by enanthic acid (Figure 7B). Among these neurons, 15 local interneurons exhibited phasic excitatory on-responses to enanthic acid (Figure 7D).

### Temporal dynamics of enanthic acid-induced activity in secondary olfactory interneurons

**Figure 8:**
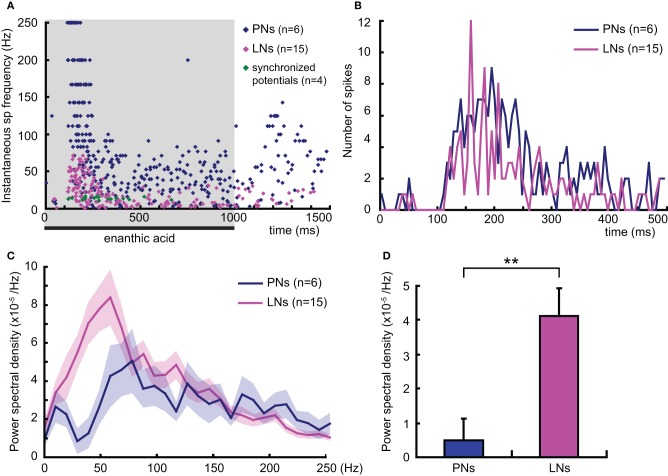
**Temporal dynamics of enanthic acid-induced action potentials of secondary olfactory inter neurons. (A)** The temporal distributions of enanthic acid-induced action potentials of projection neurons (PNs: blue dots), local interneurons (LNs: magenta dots), and synchronized potentials (green dots). Instantaneous spike frequencies calculated from six projection neurons (16 responses), 15 local interneurons (32 responses), and four VSD imaging (4 responses) were summarized. The time distributions of instantaneous spike frequencies in individual secondary olfactory neurons are available as Figure A1. The shaded area indicates the period of stimulation with enanthic acid in intracellular recording. **(B)** Time histograms of enanthic acid-induced action potentials of projection neurons (PNs: blue line) and local interneurons (LNs: magenta line). 202 spikes obtained from 6 projection neurons and of 131 spikes from 15 local interneurons during the first 500 ms period of enanthic acid stimulation were, respectively, summarized. Spikes were counted every 6 ms, which was the time resolution of VSD imaging. **(C)** Power spectral densities of the neural activities of secondary olfactory interneurons. Each power spectral density was calculated by subtracting the background in the peri-stimulus period (–500 to 0 ms) from that in the enanthic acid-stimulus periods (0–500 ms). The blue and magenta lines show the averaged power spectral density of 16 responses of 6 projection neurons and 32 responses of 15 local interneurons, respectively. The shaded areas show the standard errors of the power spectral densities. **(D)** Averaged spectral power of the enanthic acid-induced neural activities of secondary olfactory interneurons in the range of 12–18 Hz. **significantly different, *P* < 0.01; Welch's *t*-test.

**Figure A1:**
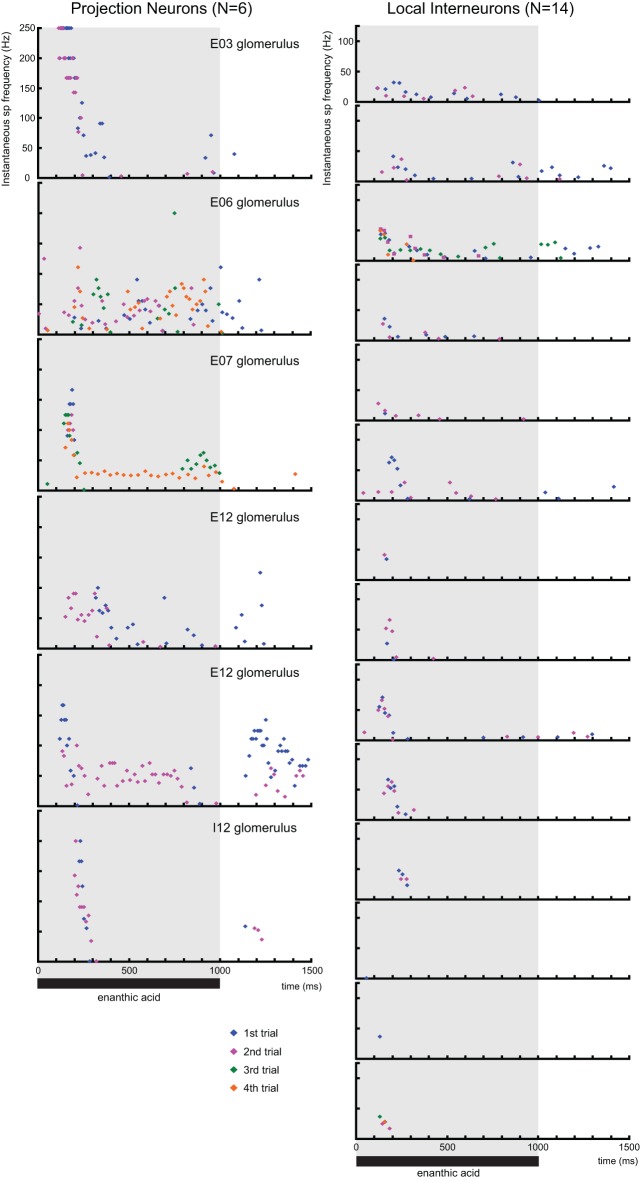
**The time distributions of instantaneous spike frequencies in individual secondary olfactory neurons.** Instantaneous spike frequencies calculated from six projection neurons (16 responses), 14 local interneurons (31 responses) are shown as raster displays. Because one local interneuron exhibited only one spike in response to enanthic acid stimuli, time distributions of instantaneous spike frequencies of the neuron have been omitted from raster displays. The shaded area indicates the period of stimulation with enanthic acid. Blue, magenta, green, and orange dots indicate action potentials during first, second, third, and fourth trials, respectively.

To analyze the temporal dynamics of enanthic acid-induced neural activities in secondary olfactory interneurons, we compared the time distributions of the enanthic acid-induced action potentials obtained by intracellular recording from secondary olfactory interneurons with the patterns of synchronized potentials obtained by VSD imaging (Figure 8). First, we summarized the time distributions of instantaneous spike frequencies calculated from 386 action potentials of six projection neurons (16 responses, blue dots in Figure 8A), 205 action potentials of 15 local interneuons (32 responses, magenta dots in Figure 8A) and 22 synchronized potentials of four VSD imaging (4 response, green dots in Figure 8A). The time distributions of instantaneous spike frequencies in individual secondary olfactory neurons are available as Figure A1. In both projection neurons and local interneurons, spike frequencies clearly increased in the period from 150 to 500 ms after the onset of enanthic acid stimulation, and those were corresponded to temporal structure of synchronized potentials. During first 500 ms period of enanthic acid, projection neurons discharged in higher frequency (median = 76.9 Hz, 25–75 percentiles = 37.0–142.9 Hz) compared with local interneurons (median = 22.7 Hz, 25–75 percentiles = 10.2–41.7 Hz) (Welch's *t*-test, *P* = 3.2 × 10^−30^). Instantaneous spike frequencies of synchronized potentials in VSD imaging were highly stable at 14 Hz in the time period (median = 14.1 Hz, 25–75 percentiles = 12.8–16.7 Hz).

VSD imaging acquired the averaged membrane potential during 6 ms period of each frame in our experiments. To elucidate the signal sources of synchronized potentials in VSD imaging, the numbers of action potentials recorded from projection neurons and local interneurons were counted for each 6 ms interval during the first 500 ms of olfactory stimulation (Figure 8B). The total numbers of action potentials are 202 spikes in projection neurons and 131 spikes in local interneurons. The spike histogram of local interneurons exhibited a zigzag shape (magenta line in Figure 8B), indicates that multiple local interneurons fired in the same time period in response to enanthic acid stimulation. For example, eight of the 15 local interneurons exhibited spike activities between 156 and 162 ms after onset of the enanthic acid stimuli (Figure 8B). In contrast, the projection neurons fired various timing one another in all phases of the olfactory responses (blue line in Figure 8B). The synchronized firing of multiple local interneurons was mainly observed in the period from 100 to 300 ms after the onset of enanthic acid stimulation, and the synchronization seems to be coincident with the temporal structure of synchronized potentials in VSD imaging. Finally, the power spectral densities of projection neurons and local inter neurons during the first 500 ms of enanthic acid stimulation were analyzed (Figure 8C). The averaged power spectral density of 15 local interneurons was significantly greater than that of six projection neurons at low frequency (10–50 Hz) (Figure 8C). A single pronounced peak was observed around 50 Hz in the power spectral density curves of local interneurons, whereas several peaks were appeared between 70 and 200 Hz in projection neurons. These results indicate that multiple local interneurons exhibit similar frequency characteristics of membrane potentials in response to enanthic acid stimulations. Especially, within the range of 12–18 Hz, which corresponds to the frequency of synchronized potentials in VSD imaging (Figure 8A), the averaged power spectral density of local interneurons was significantly greater than that of the projection neurons (Welch's *t*-test, *P* = 0.0013, Figure 8D). Taken together the results of spike histograms and power spectral densities, multiple local interneurons synchronously fire in response to enanthic acid and it might induce oscillated membrane potentials onto a particular set of glomeruli, whose amplitudes are detectable in VSD imaging.

### Paired intracellular recording from two local interneurons

**Figure 9:**
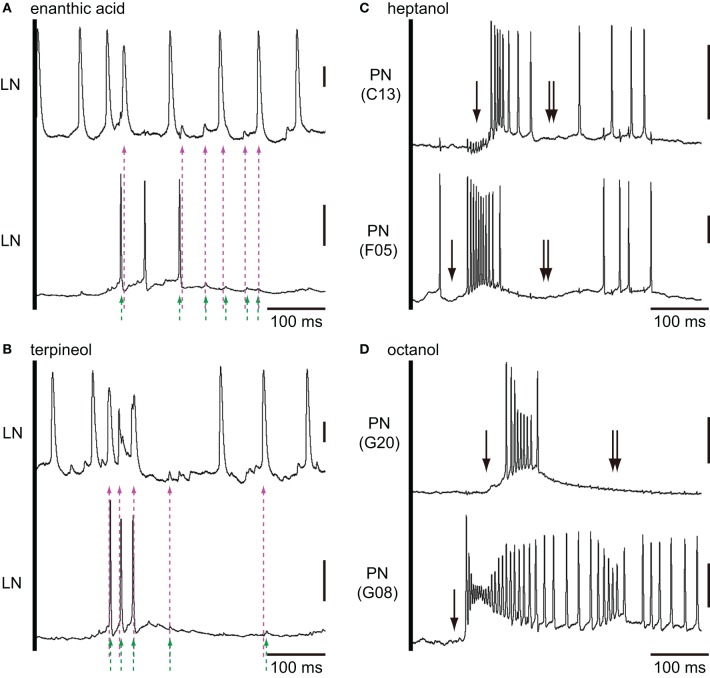
**Paired intracellular recordings from two different secondary olfactory interneurons. (A,B)** Changes of membrane potentials of a couple of local interneurons during the first 500 ms period of enanthic acid **(A)** and terpineol **(B)** stimulations. Couples of magenta and green broken arrows indicate the peaks of membrane potentials of two different neurons which appear within the 6 ms period. We judged these spikes are temporally synchronized. **(C)** Heptanol-induced excitatory responses of two different projection neurons arborizing C13 and F05 glomeruli. **(D)** Octanol-induced excitatory responses of two different projection neurons arborizing G8 and G20 glomeruli. Both couples of projection neurons exhibited strong excitatory responses to the given odorants with different latencies. Projection neurons exhibited characteristic biphasic (IPSP-EPSP) or triphasic (IPSP-EPSP-IPSP) olfactory responses. The triphasic response consists of a phasic IPSP (single arrows), followed by an EPSP involving many action potentials. And, another hyperpolarizing phase (double arrows) follows EPSP prevented the PN from firing for 100–300 ms. Vertical bars = 20 mV.

In order to examine odor-induced synchronized activities of multiple local interneurons, we performed paired intracellular recordings from two distinct secondary olfactory interneurons (Figure 9). We succeeded to record neural activities simultaneously from a couple of enanthic acid-responsive local interneurons (Figures 9A,B). Both local interneurons had neurites within glomeruli where enanthic acid-induced synchronized potentials were observed in VSD imaging (data not shown). Both neurons exhibited excitatory on-responses to enanthic acid (Figure 9A) and terpineol (Figure 9B). During enanthic acid stimulation, six peaks of action potentials or EPSPs of one neuron were synchronized to those of the other neuron within the period of 6 ms (magenta and green broken arrows; Figures 9A,B). The odor-induced synchronized activities of two local interneurons were also observed in terpineol stimulations (Figure 9B). Since all peaks of membrane potentials induced by these odorants were not synchronized, frequencies calculated from synchronized spikes (broken arrows) were lower than those calculated from all spikes in each of two neurons. The first synchronized spikes were evoked at 144 ms after onsets of enanthic acid and it was similar to the latencies of synchronized potentials observed in VSD imaging (150 ms after onsets of olfactory stimulations). On the other hand, two projection neurons arborizing in different glomeruli revealed different latencies and different temporal structures of odor-induced action potentials (Figures 9C,D). Projection neurons exhibited characteristic biphasic (IPSP-EPSP) or triphasic (IPSP-EPSP-IPSP) olfactory responses. The triphasic response consists of a phasic on-response of IPSP (black arrow in Figures 9C,D), followed by a EPSP, which gives rise to many action potentials. Another hyperpolarizing phase follows EPSP and can prevent the PN from firing for 100–300 ms (double black arrows in Figures 9C,D). We could not identify synchronized spikes between two different projection neurons except for a few spikes occurred during the EPSP phases. These results strongly support our hypothesis that the odor-induced synchronized potentials in VSD imaging reflect the odor-induced synchronized firing of multiple local interneurons.

## Discussion

High-speed optical imaging using a VSD is a valuable technique for analysis of spatio-temporal activity dynamics in the brain. In this study, VSD imaging of the cockroach AL revealed that distinct odorants induced depolarizing potentials in different sets of glomeruli. Since the depolarizing potentials induced by each odorant were temporally synchronized among glomeruli, we have termed them “synchronized potentials”. The temporal patterns of synchronized potentials appear to be odorant-specific. The spatio-temporal patterns of synchronized potentials did not change between first and second trials of the same odor stimulations. It suggests that the odor-induced spatio-temporal patterns of synchronized potentials may not be affected by small changes of odor experience and of odor concentrations. Although it requires more quantitative analysis of the effects of changes of odor concentrations and odor experience on the spatio-temporal patterns of synchronized potentials, our results suggest that odor identities are encoded as specific spatio-temporal patterns of synchronized potentials.

VSD imaging allowed us to monitor simultaneously both spatial and temporal patterns of overall summed membrane potentials of neural assembles, and we were able to demonstrate odorant-specific synchronized potentials in the AL. Odor-specific spatial activity patterns among glomeruli in vertebrates and invertebrates have been demonstrated by optical imaging using a calcium-sensitive dye (Galizia et al., 1999; Rubin and Katz, 1999; Sachse et al., 1999; Sachse and Galizia, 2002; Wang et al., 2003; Soucy et al., 2009). However, calcium signals are not suitable for temporal analysis because of their slowly decaying phase (Galizia et al., 2000). To investigate the temporal dynamics of the olfactory responses of neural circuits, local field potential recording is often used (Laurent and Davidowitz, 1994; Laurent et al., 1996; MacLeod and Laurent, 1996; Stopfer et al., 1997; Christensen et al., 2003). In honeybees, odorant-specific temporal patterns of local field potentials in the AL play critical roles in fine discrimination of odorants (Stopfer et al., 1997). However, it is still controversial whether the oscillations of the local field potentials reflect synchronized firing across a global neural population of the AL or the summation of activities of neurons in the immediate vicinity of the recording electrodes (Christensen et al., 2003). VSD imaging of the cockroach AL could record changes of membrane potentials in 454 pixels covered anteriorly located 47 glomeruli at same time with better temporal resolution (6 msec) than calcium imaging. Therefore, odor-induced spatio-temporal activity patterns of synchronized potentials detected by VSD imaging may pave the way to clarification of the neural basis of olfactory processing in the primary olfactory centers of animals.

We demonstrated that odor-evoked synchronized potentials are not propagated whole AL, but are confined to the glomeruli. This result is consistent with the results of previous VSD imaging and local field potential recording studies from primary olfactory centers in insects and vertebrates. In bumblebees, VSD recording from the AL also revealed that odor-evoked oscillatory signals occurred in limited regions that corresponded to one or a few glomeruli (Okada and Kanzaki, 2001). In moths, simultaneous local field potential recordings from two distinct sites in the AL revealed that different odors evoke different spatial patterns of local field potential in the AL (Christensen et al., 2003). VSD imaging studies using the turtle olfactory bulb also showed similar results (Lam et al., 2000, 2003; Zochowski and Cohen, 2005). On the other hand, there are inconsistencies in the characteristics of the oscillatory signals among animals. VSD imaging of bumblebees and turtles did not detect odor-specific temporal patterns in the oscillatory signals, while odor-specific temporal patterns of synchronized potentials were detected by VSD imaging of the cockroach AL in this study, and by local field potential recordings from locusts (Laurent et al., 1996) and fruits flies (Tanaka et al., 2009).

### Neural basis of the synchronized potentials

The signal intensity of VSD imaging is lower than that of calcium imaging. Therefore, VSD imaging has been barely used for the analysis of odor representations in the primary olfactory centers, in spite of its advantages for temporal analysis of neural signals. Only in the primary olfactory centers of the bumblebee (Okada and Kanzaki, 2001) and the turtle (Lam et al., 2000, 2003; Zochowski and Cohen, 2005), VSD imaging has revealed temporal patterns of odor-induced oscillatory potentials. However, odor-induced spatial patterns of VSD signal could not be identified at the level of individual glomeruli in these animals, and it makes difficult to compare the VSD images between individuals. In the cockroach AL, all glomeruli, especially glomeruli of the postero-ventral group, have been identified completely (Watanabe et al., 2010, 2012), and this has enabled us to identify glomeruli where enanthic acid-induced synchronized potentials were evoked. Furthermore, we succeeded in comparing enanthic acid-induced temporal activity patterns of synchronized potentials occurred in a particular set of glomeruli with those of action potentials in secondary olfactory interneurons innervating these glomeruli. Thus, the cockroach AL is a suitable system for analyzing neural network activities, particularly “synchronization”.

**Figure 10:**
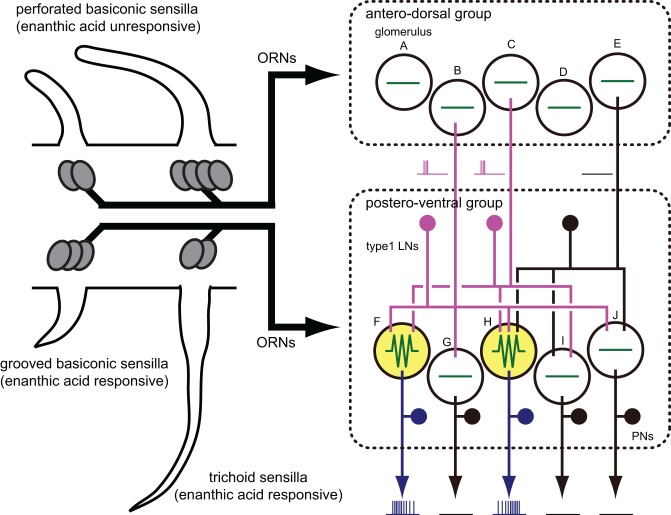
**A model of the neural basis of enanthic-acid processing.** On the antennal surface of the cockroach, three distinct morphological types of olfactory sensilla have been identified; perforated basiconic, trichoid and grooved basiconic sensilla. Among these sensilla, enanthic-acid selectively received by olfactory receptor neurons (ORNs) housed in trichoid and grooved basiconic sensilla (Fujimura et al., 1991). Our anatomical study revealed that ORNs in trichoid and grooved basiconic sensilla selectively project to glomeruli of the postero-ventral group (Watanabe et al., 2012). Therefore, information of enanthic acid is mainly represented in glomeruli of the postero-ventral group. In fact, VSD imaging revealed that enanthic acid generated synchronized potentials only in glomeruli of the postero-ventral group. The cockroach AL consists of about 300 GABAergic local interneurons, 300 projection neurons, and 205 glomeruli (Boeckh and Tolbert, 1993; Watanabe et al., 2010). In this model, we simplify the cockroach AL as 3 GABAergic local interneurons (type-1 LNs), five uniglomerular projection neurons (PNs) and 10 glomeruli (the antero-dorsal group glomeruli A–E, the postero-ventral group glomeruli F–J). Enanthic acid induces excitatory responses in some of the GABAergic local interneurons (magenta lines) and the odor-induced action potentials are temporally synchronized across these local interneurons. Since each glomerulus receives innervations from about 25 local interneurons in the cockroach AL (Boeckh and Tolbert, 1993), convergence of the synchronized firing of multiple local interneurons onto a particular set of glomeruli (glomeruli F and H colored yellow) induces strong synchronized potentials in these glomeruli, which are detectable in VSD imaging (green lines). In contrast, the glomeruli (shown by glomeruli A–E, G, I, and J) that receive inputs from a small number of activated local interneurons, remain silent. Since enanthic acid-responsive projection neurons (blue lines) have dendritic arborizations in the glomeruli where the synchronized potentials are evoked (Figure 6), the spatial activity pattern of projection neurons might be shaped by inhibitory inputs from synchronized local interneurons.

We proposed the hypothesis that enanthic acid-induced synchronized potentials recorded by VSD imaging were caused by simultaneous firing of multiple local interneurons (Figure 10 right) for the following reasons: (1) The odor-induced synchronized potentials never occurred in the region corresponding to the antennal nerve. In addition, enanthic acid evoked the synchronized potentials in the dorsal soma cluster of secondary olfactory interneurons (Figure 5A, Distler and Boeckh, 1997a,b). From these observations, we estimate that enanthic acid-induced synchronized potentials were generated by activities of secondary olfactory interneurons, projection neurons and/or local interneurons; (2) In the cockroach AL, most of the glomeruli are innervated by single uniglomerular projection neurons (Nishino et al., 2011, 2012). VSD imaging revealed that odor-induced VSD signals were temporally synchronized across several glomeruli. However, our intracellular recordings revealed that enanthic acid-responsive projection neurons arborizing five different glomeruli exhibited different latencies and temporal dynamics of action potentials (Figure 6). In addition, paired intracellular recording revealed that odor-induced action potentials of two different projection neurons were not synchronized (Figure 9); and (3) All of our identified enanthic acid-responsive local interneurons possessed dendritic arborizations within the glomeruli where synchronized potentials were evoked by the odor in VSD imaging. These local interneurons exhibited a phasic on-response to enanthic acid (Figures 7, 8). Paired intracellular recording from two local interneurons readily revealed that a part of enanthic acid-induced action potentials were temporally synchronized each other neurons (Figure 9). In the cockroach, about 25 local interneurons converge onto a single glomerulus (Boeckh and Tolbert, 1993). Therefore, enanthic acid should generate strong synchronized depolarizing potentials in a particular set of glomeruli where multiple enanthic acid-responsive local interneurons are converging (Figure 10 right). Taken together these results, VSD imaging appears to detect the enanthic acid-induced synchronized firing of multiple local interneurons.

### Spatial activity patterns of glomeruli

All recorded enanthic acid-responsive projection neurons have dendrites within the glomeruli where enanthic acid generated synchronized potential (Figure 6). This result indicates that enanthic acid-induced synchronized potentials, which appear to be mediated by synchronized firing of the multiple local interneurons, shape the spatial activity patterns of projection neurons (Figure 10 right). The morphological features of the oligoglomerular projections of all local interneurons identified in this study matched those of so-called “type-1 local interneurons” (Husch et al., 2009a,b), which are known to be GABAergic (Husch et al., 2009b). Consistent with our results, a functional imaging study of the fruit fly AL reported odor-specific spatial patterns of GABA release (Ng et al., 2002). In addition, calcium imaging of dendrites of projection neurons in the honeybee AL revealed that application of a GABA receptor antagonist to the AL increased the number of glomeruli activated by olfactory stimulation (Sachse and Galizia, 2002). Thus, odor-specific synchronized activities of GABAergic local interneurons and subsequent GABA release in particular sets of glomeruli are involved in the spatial representation of odor-identities in postsynaptic projection neurons (Figure 10 right).

VSD imaging partially revealed functions of glomerular clusters in the AL; citral and pentanol induced synchronized potentials in the antero-dorsal group of glomeruli, whereas enanthic acid generated synchronized potentials in glomeruli of the postero-ventral group. Recently, we found that the antero-dorsal group glomeruli exclusively receive olfactory inputs from the antennal perforated basiconic sensilla, whereas the postero-ventral group glomeruli receive sensory inputs from trichoid sensilla or grooved basiconic sensilla (Figure 10 left; Watanabe et al., 2012). In the cockroach, comprehensive electrophysiological recordings from antennal olfactory sensilla classified olfactory receptor neurons into several cell groups on the basis of similarities of response spectra (Fujimura et al., 1991). Pentanol is processed by olfactory receptor neurons housed in perforated basiconic sensilla but not those in trichoid and grooved basiconic sensilla. On the other hand, olfactory receptor neurons identified in grooved basiconic and trichoid sensilla exhibits high responsiveness to enanthic acid (Figure 10 left; Fujimura et al., 1991). In addition, terpenoid molecules, such as a citral, are specific ligands of olfactory receptor neurons in the perforated basiconic sensilla (Sass, 1978; Fujimura et al., 1991). Therefore, odorant-specific spatial activity patterns of synchronized potentials are highly correlated with the projection patterns of receptor neurons and with the olfactory response spectra which are also associated with morphological types of sensilla (Figure 10). On the other hand, at the levels of individual glomeruli, there are inconsistencies in spatial patterns of glomeruli between sensillar projections and VSD imaging; olfactory receptor neurons in trichoid sensilla projected to both enanthic acid-responsive (E06, E07, and E12 glomeruli) and enanthic acid-unresponsive glomeruli (E11, E13, and E09 glomeruli) in VSD imaging (Watanabe et al., 2012). It suggests that synchronized potentials mediated by multiple GABAergic local interneurons enhance the contrast of spatial activity pattern of glomeruli. Additional experiments are needed to determine the odor-induced spatial activity patterns of glomeruli in the cockroach AL.

### Temporal structures of olfactory responses

Enanthic acid typically induced phasic excitatory on-responses in the GABAergic type-1 local interneurons and multiple local interneurons synchronously fired in response to the odor. In contrast, paired intracellular recording revealed that odor-induced action potentials of two different projection neurons were not synchronized each other. These results suggest that the timing of action potential firing in projection neurons may not be phase-locked to the synchronized firing of local interneurons. In the cockroach, projection neurons usually exhibited biphasic (IPSP-EPSP) or triphasic (IPSP-EPSP-IPSP) responses during the first 500 ms period of olfactory stimulation. Since type-1 local interneurons make GABAergic inhibitory synapses onto a projection neuron in each glomerulus (Distler et al., 1998), the odor-induced IPSP phases of projection neurons seems to be mediated by GABAergic local interneurons. In fact, intracellular recordings from projection neurons of moths (Christensen et al., 1998), locusts (MacLeod and Laurent, 1996), and fruits flies (Wilson and Laurent, 2005) all exhibit similar IPSP-EPSP complex of olfactory responses. In these animals, GABA antagonists abolished hyperpolarizing phases but not depolarizing phase (MacLeod and Laurent, 1996; Christensen et al., 1998; Wilson and Laurent, 2005; Tanaka et al., 2009).

In this study, we were unable to detect the spike activities of type-1 local interneurons corresponding to the longer decay phases of synchronized potentials induced by pentanol and citral. Unlike the synchronized potentials induced by enanthic acid, pentanol, and citral generated several amplitudes of depolarizing potential during the longer decay phase. It suggests that VSD signals induced by these odorants reflect total voltage fluctuation of several neural elements, such as olfactory receptor neurons, projection neurons, and the other types of local interneurons, at the level of an entire glomerulus. In fact, during pre-stimulus period, VSD imaging detected depolarizing potentials which are not synchronized across pixels. Since local interneurons rarely exhibited spontaneous activities (Figure 7), it appears to reflect the activities of the other neural elements. In particular, pentanol and citral induced different temporal structures of synchronized potentials in the two common pixels (Figure 3). In the cockroach, another type of local interneuron, namely type-2 local interneuron, has been morphologically and physiologically characterized (Husch et al., 2009a,b). Type-2 local interneurons have neurites in almost all glomeruli (panglomerular projection) and exhibit long-lasting excitatory responses with different temporal activity patterns in response to the different odorants (Husch et al., 2009a,b). To elucidate the temporal coding of the odor-identities, our next step will be to study comprehensively the odor-induced synchronized activities of the other neural elements composing each glomerulus using paired intracellular recording technique.

Both the oligoglomerular local interneurons, corresponding to type-1 local intereneurons in the cockroach, and panglomerular local interneurons, corresponding to type-2 local interneurons, have been identified in fruit flies (Okada et al., 2009; Seki et al., 2010) and moths (Seki and Kanzaki, 2008). It suggests that two distinct olfactory pathways in the AL are evolutionary conserved. In the fruit fly, however, synchronized firing of the oligoglomerular local interneurons shape the longer decay phase of local field potential oscillation (Wilson and Laurent, 2005; Tanaka et al., 2009). Thus, oligoglomerular local interneurons in the cockroach appear to have distinct functions to those in the fruit fly. The cockroach is advantageous as an animal for study of olfactory processing because a large amount of anatomical and electrophysiological data on single secondary olfactory neurons has been accumulated (Malun et al., 1993; Strausfeld and Li, 1999; Husch et al., 2009a,b; Nishino et al., 2011, 2012). By combining electrophysiological recording and staining of single or multiple olfactory interneurons with optical recordings from neural assemblies composing individual glomeruli, we aim to accurately and comprehensively develop a neural basis for odor representations in the insect olfactory system.

## Conflict of interest statement

The authors declare that the research was conducted in the absence of any commercial or financial relationships that could be construed as a potential conflict of interest.
